# Validation of the REduction of Atherothrombosis for Continued Health (REACH) prediction model for recurrent cardiovascular disease among United Arab Emirates Nationals

**DOI:** 10.1186/s13104-020-05331-8

**Published:** 2020-10-19

**Authors:** Saif Al-Shamsi, Romona D. Govender

**Affiliations:** 1 Department of Internal Medicine, College of Medicine and Health Sciences, United Arab Emirates University, Al Ain, United Arab Emirates; 2 Department of Family Medicine, College of Medicine and Health Sciences, United Arab Emirates University, P.O. Box 17666, Al Ain, United Arab Emirates

**Keywords:** Calibration, Discrimination, External validation, Prediction model, Recurrent cardiovascular disease, United arab emirates

## Abstract

**Objectives:**

Prediction models assist health-care providers in making patient care decisions. This study aimed to externally validate the REduction of Atherothrombosis for Continued Health (REACH) prediction model for recurrent cardiovascular disease (CVD) among the Emirati nationals.

**Results:**

There are 204 patients with established CVD, attending Tawam Hospital from April 1, 2008. The data retrieved from electronic medical records for these patients were used to externally validate the REACH prediction model. Baseline results showed the following: 77.0% were men, 69.6% were diagnosed with coronary artery disease, and 87.3% have a single vascular bed involvement. The risk prediction model for cardiovascular mortality performed moderately well [*C*-statistic 0.74 (standard error 0.11)] in identifying those at high risk for cardiovascular death, whereas for recurrent CVD events, it performed poorly in predicting the next CVD event [*C*-statistic 0.63 (standard error 0.06)], over a 20-month follow-up. The calibration curve showed poor agreement indicating that the REACH model underestimated both recurrent CVD risk and cardiovascular death. With recalibration, the REACH cardiovascular death prediction model could potentially be used to identify patients who would benefit from aggressive risk reduction.

## Introduction

Prediction models assist health-care providers in making important daily patient care decisions by population screening, assessing responses to treatment, intensifying management, and motivating patients toward behavior change [1]. In addition, prediction models help risk-stratify patients into low, moderate, high, or very high-risk groups for appropriate prevention and management strategies [1, 2]. Those deemed high- or very-high-risk individuals need far more aggressive management and monitoring [2]. Having the diagnosis of cardiovascular disease (CVD) puts patients at an increased risk for a recurrent CVD event [3]. In a previous study, ischemic heart disease was projected to increase by 138% and 158% for women and men, respectively, from 1990 to 2020 in the Middle East [4]. Although alarming, these figures are likely to increase or decrease in the future as the pattern of CVD risk continues to change in most of the developing countries [4, 5].

Wilson et al. developed a risk prediction model for recurrent CVD events or cardiovascular death, with participants from the REduction of Atherothrombosis for Continued Health (REACH) Registry [6]. The researchers conducted and internally validated their risk prediction tool on 68,000 patients from 44 countries. These include patients from the Middle East [7], thus making it the most globally inclusive, ethnically diverse, and geographically extensive registry of CVD patients. The patient population from the Middle East made up 1.25% (850) of the total sample. It included countries such as Saudi Arabia [198 participants (0.29%)] and the United Arab Emirates (UAE) [149 participants (0.22%)] [7]. This study revealed that the risk for recurrent CVD was high among the Middle Eastern population [6]. Therefore, based on the geographical region and availability of similar data as defined in the REACH model, it played a significant role in our decision to externally validate this risk tool in our clinical setting. Empirical evidence shows that models tend to perform better when internally validated and not as well in other cohorts in which the model may be applied [8–11]. This illustrates the need for external validation (discrimination and calibration) among patients exclusively from the UAE in order to assess its fitness for recurrent CVD risk prediction. Thus, this study aimed to externally validate the REACH prediction tool among UAE nationals with established CVD.

## Main text

### Methods

#### Study design, data source, and study cohort

This is an external validation study. The study site was Tawam Hospital’s outpatient clinics. Tawam Hospital is the largest publicly funded teaching hospital in Al Ain providing health care exclusively to UAE nationals. It is affiliated with the College of Medicine and Health Sciences, UAE University. UAE nationals are defined as the citizens of the UAE.

All UAE nationals enrolled in this study were diagnosed with a CVD event, who were visiting the outpatient clinics over a 9-month period in 2008. The recruitment was done retrospectively using electronic medical records (EMRs). The inclusion criteria for this study were matched to that of the developmental REACH cohort [6]. It included patients aged 45 years and older with established CVD. Patients with CVD are defined as patients with cerebrovascular disease, coronary artery disease (CAD), or peripheral vascular disease (PVD), based on the documented diagnosis by a physician. There were 206 patients who met the inclusion criteria, but 2 patients with missing data at baseline were excluded.

#### Baseline characteristics

Baseline data were obtained from the EMRs. The sociodemographic and clinical variables were as follows: age, sex, body mass index (BMI), smoking status, number of vascular beds involved, geographic region (in this case, the UAE), and history of diabetes mellitus, congestive heart failure (CHF), atrial fibrillation (AF), a baseline cardiovascular event (defined as a documented diagnosis of myocardial infarction or cerebrovascular disease) occurring in the previous 24 months, antihypertensive and lipid-lowering medications, and acetylsalicylic acid therapy. CHF and AF were not defined as a CVD event by Wilson et al. [6] In this study, smokers had either a history of smoking or currently smoking, and BMI was stratified as < 20, 20–30, and > 30 kg/m^2^. Diabetes mellitus was established by a documented clinician’s diagnosis. Patients were classified as having hypertension, when they have a diastolic blood pressure ≥ 90 mmHg or systolic blood pressure ≥ 140 mmHg or are on antihypertensive medications.

#### Endpoints

Although participants were followed up for 24 months, the cutoff used was 20 months as defined by Wilson et al. [6] Cardiovascular death was defined as the occurrence of a fatal MI or fatal stroke, whereas a recurrent CVD event was the occurrence of cardiovascular death, a cerebrovascular disease, or a myocardial infarction (MI) event.

#### Data analysis

Baseline characteristics of the validation cohort were compared to the developmental cohort. Student’s t-test was used for continuous variables, while Fisher’s exact test or chi-square was used for categorical variables. The time between study enrolment and the first occurrence of a recurrent CVD event during the follow-up period was defined as the months at risk for developing the endpoints.

The probability of each participant developing each endpoint was calculated by applying the regression coefficients of the original Cox regression models for recurrent CVD and cardiovascular death in the developmental study. External validation is performed by evaluating the REACH model’s predictive performance by calibration and discrimination [12]. Calibration and discrimination were evaluated at 20 months of follow-up. Discrimination is defined as the capability to correctly differentiate between two participants based on a risk score, i.e., one who will not develop the endpoint and one who will over the follow-up duration [13]. On the other hand, calibration is defined as the degree of agreement between the risks predicted by the model and the observed frequencies of the outcomes under study. Discrimination was calculated using Harrell’s *C*-statistic with standard error (SE) [14]. Calibration was assessed graphically by comparing the predicted 20-month events with the observed events, grouped by quintiles of predicted probabilities. The R software version 3.5.2, using the packages “rms” and “riskRegression,” was used to analyze our data.

### Results

Table 1 shows the baseline characteristics of both the developmental [6] and validation cohorts. The validation sample size included 204 participants, of which 77% were men, with a mean age of 67 years at baseline. Participants with CAD, cerebrovascular disease, and PVD together formed the CVD burden at 69.6, 38.2, and 6.4%, respectively. On the other hand, those with one vascular bed involvement were the most prevalent at 87.3%. Among the study cohort, 32% had experienced a CVD event in the previous year, whereas 10.3 and 6.4% had CHF and AF, respectively. Almost 26% were smokers, and approximately 63% were patients with diabetes mellitus. A very high percentage were on antihypertensive medications (93.6%), lipid-lowering medications (85.3%), and only 52.5% on acetylsalicylic acid (commonly known as aspirin).

**Table 1:** Baseline characteristics comparison between the REACH and external validation cohorts

Baseline characteristics	REACH cohort (n = 33 419)	External validation cohort (n = 204)	*P* value
Men, n (%)	22,357 (66.9)	157 (77.0)	0.002
Age, year, mean ± SD	68.4 ± 10.1	67.3 ± 9.1	0.121
Smoking, n (%)	4,879 (14.6)	52 (25.5)	< 0.001
Diabetes mellitus, n (%)	12,332 (36.9)	129 (63.2)	< 0.001
BMI	27.7 ± 5.4	26.9 ± 5.1	0.035
BMI > 30 kg/m^2^, n (%)	9,090 (27.2)	52 (25.5)	0.584
BMI < 20 kg/m^2^, n (%)	1,270 (3.8)	16 (7.8)	0.003
Systolic BP, mm Hg, mean ± SD	136.8 ± 19.3	136.3 ± 19.3	0.712
Diastolic BP, mm Hg, mean ± SD	78.2 ± 11.2	75.1 ± 12.2	< 0.001
Cholesterol, mg/dL	191.4 ± 45.9	167.8 ± 49.9	< 0.001
Atrial fibrillation, n (%)	3,910 (11.7)	13 (6.4)	0.018
Cardiovascular burden, n (%)			
Coronary artery disease	24,195 (72.4)	142 (69.6)	0.374
Cerebrovascular disease	11,296 (33.8)	78 (38.2)	0.182
Peripheral vascular disease	4,979 (14.9)	13 (6.4)	< 0.001
Number of vascular beds			
One	27,003 (80.8)	178 (87.3)	0.020
Two	5,748 (17.2)	23 (11.3)	0.025
Three	668 (2.0)	3 (1.5)	0.591
CVD event^a^ in past 12 months	10,527 (31.5)	65 (31.9)	0.911
CHF	5,080 (15.2)	21 (10.3)	0.051
Cardiovascular treatment, n (%)			
Lipid-lowering agents	23,126 (69.2)	174 (85.3)	< 0.001
Hypertension treatment	30,411 (91.0)	191 (93.6)	0.191
Acetylsalicylic acid	23,895 (71.5)	107 (52.5)	< 0.001
Geographic region: Middle East	434 (1.3)	204 (100.0)	< 0.001

*REACH* REduction of Atherothrombosis for Continued Health, *BMI* body mass index, *BP* blood pressure, *CVD* cardiovascular disease, *CHF* congestive heart failure

^a^A cardiovascular event defined as a documented diagnosis of myocardial infarction or cerebrovascular disease

Additional file 1: Table S1 outlines the number of recurrent CVD events and cardiovascular deaths observed with almost 4% having a non-fatal MI and 3% a fatal MI.

#### Discrimination

*C*-statistic was used to assess the association between the individuals from the validation sample and the developmental sample over 20 months. In the model performance analysis for recurrent CVD events, the *C*-statistic was 0.63 (SE 0.06) for a 20-month risk. Thus, the model performed poorly in predicting the next CVD event. On the other hand, for cardiovascular death, discrimination for a 20-month risk prediction was moderate, with a *C*-statistic of 0.74 (SE 0.11). Thus, the model performed moderately well to identify patients at the highest risk for cardiovascular mortality.

#### Calibration

**Fig. 1:**
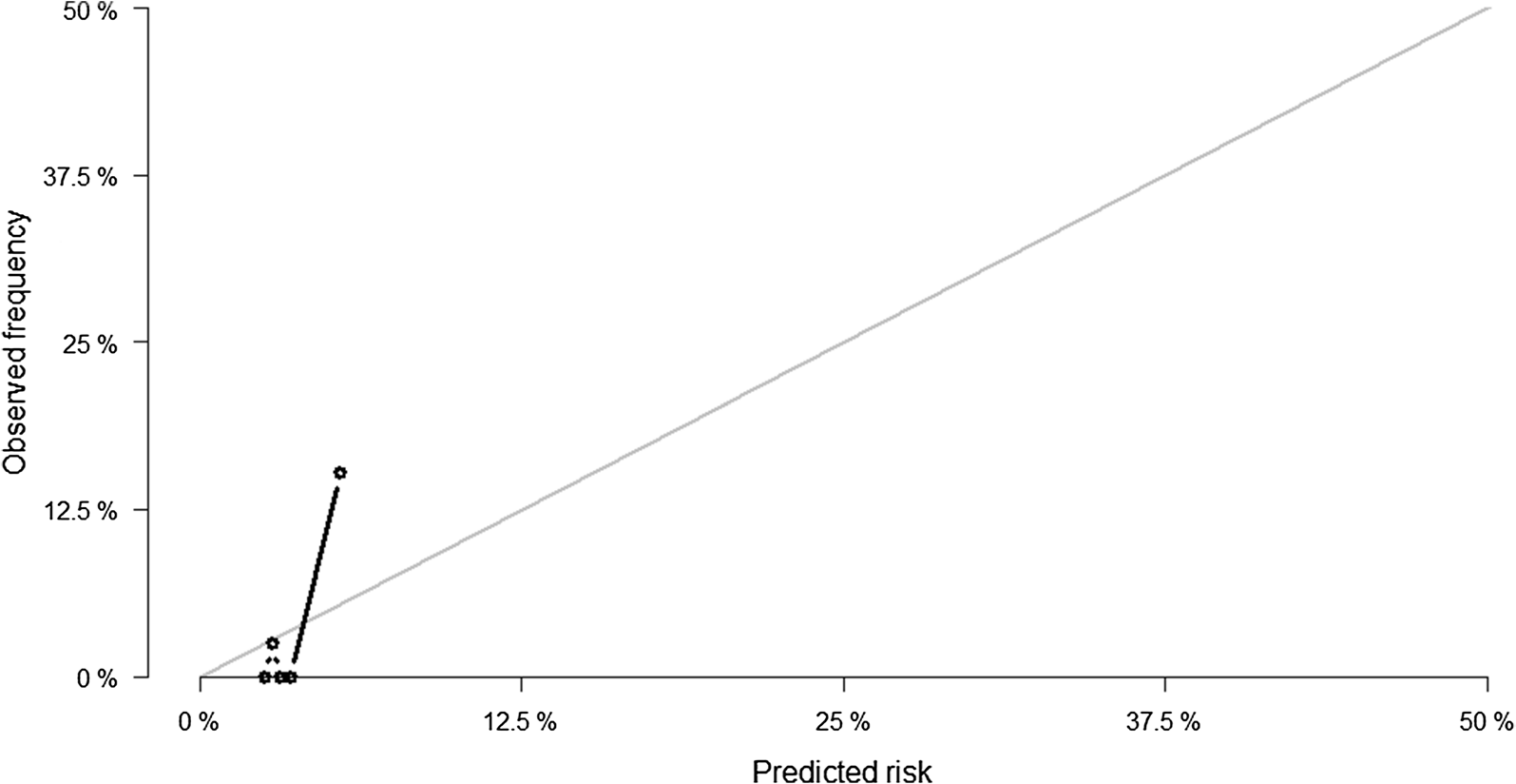
Calibration plot by quintiles of predicted and observed 20-month recurrent CVD events for the REACH prediction model. *CVD* cardiovascular disease, *REACH* REduction of Atherothrombosis for Continued Health

**Fig. 2:**
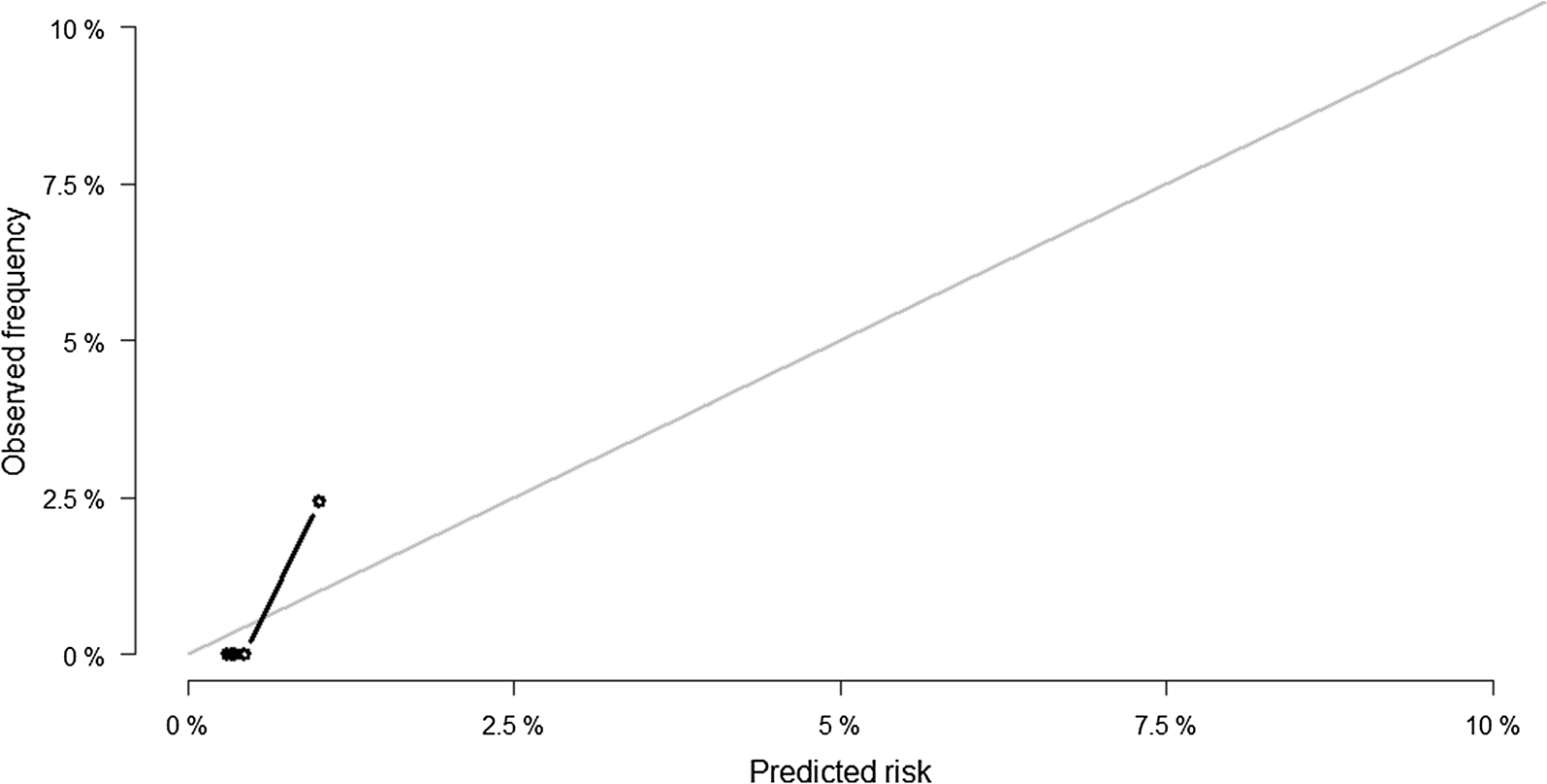
Calibration plot by quintiles of predicted and observed 20-month cardiovascular death events for the REACH prediction model. *CVD* cardiovascular disease, *REACH* REduction of Atherothrombosis for Continued Health

The calibration curves for predicting the 20-month survival probability for recurrent CVD events and cardiovascular death are shown in Figs. 1 and 2, respectively. The calibration plot showed poor agreement between predicted recurrent CVD events and observed 20-month recurrent CVD event probabilities. In addition, the REACH model underestimated both the risk of recurrent CVD events and cardiovascular death.

#### Predicting death using the risk prediction model: an example

Developed by Wilson et al. [6], the recurrent risk prediction tool included the sum of numerical values assigned to the risk factors present in a patient to predict the next cardiovascular event. An arbitrary case in practice of a 45-year-old UAE man, with a history of diabetes mellitus, smoking, CHF, BMI > 20 kg/m^2^, and involvement of a single vascular bed, would have a 20-month risk of cardiovascular death of approximately 4.9%.

### Discussion

This study represents the first external validation of the REACH prediction model for recurrent CVD exclusively for UAE nationals. The external validation results performed moderately well in discriminating those participants likely to die from a recurrent CVD event from those who were more likely to survive, whereas the calibration performed poorly by underestimating the cardiovascular mortality risk, over a 20-month period of follow-up. This outcome is not unusual as research has shown that prognostic tools developed to predict mortality are less than perfect [15]. Moreover, CVD risk prediction tools show a reduced accuracy when validated in new cohorts, mainly due to the differences in characteristics between the developmental and validation cohorts [16].

The notable differences between the developmental cohort and our study cohort could explain the underestimation of predicted risk of recurrent CVD and cardiovascular death seen in this study. In this study, patients were more likely to smoke and have diabetes mellitus, and only half were on aspirin therapy. Furthermore, the distribution of participants in the developmental model was across 44 countries. The types of included clinical practices were far more extensive, covering both urban and rural areas [7]. Despite this, with recalibration, this model could have the potential to help clinicians identify individuals at the greatest short-term risk and, therefore, benefit from intense follow-up and management.

### Conclusion

Using the REACH model, recurrent CVD risk prediction was found to demonstrate a modest discriminative capacity for predicting death, with an underestimation of CVD risk in the short term. Although the current application may fail to identify high-risk individuals, with recalibration, it may still be of value for close monitoring, intensification of risk factor management, and tertiary prevention. Following international guidelines for CVD, management should be the ultimate goal of primary care physicians and specialists alike.

## Limitations

The accuracy of documentation has to be accepted when using retrospective data. Differences in results may be attributed to a homogeneous case mix in our validation set. Also, the relatively low number of events may have affected the reliability of the analysis [17]. Despite the small sample size and recruitment of UAE nationals from a single center, the performance with the new data may be sufficient for the model to be potentially useful in our population until further validation studies are conducted. We encourage other researchers to also externally validate the REACH risk prediction tool, particularly with respect to recurrent CVD events. Nevertheless, this is the first study that externally validated a model for clinicians treating an increasing number of high-risk patients with CVD in this region.

## Supplementary information

**Additional file 1: Table S1.** Frequency of recurrent CVD events^a^ and cardiovascular death^b^.

## Data Availability

The datasets used and/or analysed during the current study are available from the corresponding author on reasonable request.

## Abbreviations

AF: Atrial fibrillation
BMI: Body mass index
BP: Blood pressure
CVD: Cardiovascular disease
CAD: Coronary artery disease
CHF: Congestive heart failure
CI: Confidence intervals
EMRs: Electronic medical records
PVD: Peripheral vascular disease
SE: Standard error
UAE: United Arab Emirates
