# Supplementary material for: Validation of the REduction of Atherothrombosis for Continued Health (REACH) prediction model for recurrent cardiovascular disease among United Arab Emirates Nationals
Source: BMC Res Notes. 2020 Oct 19;13:484. doi: 10.1186/s13104-020-05331-8 (PMC7574310; doi:10.1186/s13104-020-05331-8)
Supplement: Supplementary file 1 — Additional file 1: Table S1. Frequency of recurrent CVD eventsa and cardiovascular deathb. [file 13104_2020_5331_MOESM1_ESM.docx]

| **Table S1. Frequency of recurrent CVD events^a^ and cardiovascular death^b^** | | |
| --- | --- | --- |
|  | | **Total (n = 204), n (%)** |
| **Myocardial infarction** | | 8 (3.9) |
|  | **Cerebrovascular disease** | 12 (5.9) |
|  | **Fatal myocardial infarction** | 6 (2.9) |
| **Fatal stroke** | | 0 (0.0) |

CVD, cardiovascular disease

^a^Defined as cardiovascular death, cerebrovascular disease, or myocardial infarction

^b^Defined as fatal myocardial infarction or fatal stroke
